# A trade-off between cognitive and physical performance, with relative preservation of brain function

**DOI:** 10.1038/s41598-017-14186-2

**Published:** 2017-10-20

**Authors:** Daniel Longman, Jay T. Stock, Jonathan C. K. Wells

**Affiliations:** 1 Department of Archaeology and Anthropology, University of Cambridge, Cambridge, CB2 3QG UK; 2 Department of Anthropology, University of Western Ontario, Ontario, Canada; 3 Childhood Nutrition Research Centre, UCL Institute of Child Health, London, WC1N 1EH UK

## Abstract

Debate surrounds the issue of how the large, metabolically expensive brains of *Homo sapiens* can be energetically afforded. At the evolutionary level, decreased investment in muscularity, adiposity and the digestive tract allow for a larger brain. Developmentally, high neo-natal adiposity and preferential distribution of resources to the brain provide an energetic buffer during times of environmental stress. Through an experimental design, we investigated the hypothesis of a trade-off involving brain and muscle at the acute level in humans. Mental performance was measured by a free-recall test, and physical performance by power output on an indoor rowing ergometer. Sixty-two male student rowers performed the two tests in isolation, and then again simultaneously. Paired samples *t*-tests revealed that both power output and mental performance reduced when tested together compared to in isolation (*t*(61) = 9.699, p < 0.001 and *t*(61) = 8.975, p < 0.001). Furthermore, the decrease in physical performance was greater than the decrease in mental performance (*t*(61) = −2.069, p = 0.043). This is the first investigation to demonstrate an acute level trade-off between these two functions, and provides support for the selfish brain hypothesis due to the relative preservation of cognitive function over physical power output. The underlying mechanism is unclear, and requires further work.

## Introduction

### Evolutionary and developmental implications of enhanced encephalization

The development of an enlarged and elaborated brain is considered a defining characteristic of human evolution^1^. The evolution of the *Homo* clade has been accompanied by significant encephalization^2,3^. This facilitated the development of more complex social strategies^4,5^, more effective food acquisition^6^ and the ability to solve ecological problems through innovative means^7^. Each of these characteristics may have increased survival and reproductive success, giving a greater life expectancy at the age of first reproduction^8^.

While the benefits of encephalization are numerous, the brain imposes significant metabolic costs on both the individual^9–11^. High levels of energetic expenditure are necessitated by the brain’s responsibility for regulating the body’s energy supply and controlling the function of many peripheral organs^12^. These functions require intense neuronal activity, giving the brain the highest metabolic demand relative to size of all organs^13^.

The question of how larger brains can be metabolically afforded has remained a prominent problem in human evolution^11,14–17^. Life history theory states that as energy availability is finite, an organism has a limited energy budget. Energy allocated to one function cannot be used for another. Energy savings in other organs or tissues could allow for energetic diversion to the brain, without the need to increase overall metabolic expenditure^11,18^. Such a trade-off has been proposed with both digestive tract development^17^ and adiposity^19^.

### Meeting the brain’s metabolic requirements

The immediate metabolic costs of the brain depend on its activation state. While the metabolic rate is low during sleep^20^ increased energy consumption has been observed in response to a mental task^21^, and following somatosensory, olfactory, visual and auditory stimulation^22–27^. The adult brain almost exclusively derives its energy from the metabolism of glucose^28^. This, coupled with its high energetic demand, ensure that the brain metabolises the most glucose of any organ^29,30^. The brain, however, is unable to store significant amounts of energy and hence buffer its high yet variable metabolic demand^31^. As such, the body is required to supply glucose to the brain quickly and effectively. The ‘Selfish Brain Hypothesis’^12^ posits that the brain prioritises its own glucose needs over those of the peripheral organs, such as skeletal muscle.

### Skeletal muscle and encephalization

Skeletal muscle mass is an expensive tissue to maintain, accounting for approximately 20% of human male BMR^32,33^, and may be compromised to partially offset the brain’s high energy costs^11,34^. An adaptation to reduce muscle mass would thereby reduce metabolic demand, allowing for a reallocation of energy towards the central nervous system^35^. The glucose demands of skeletal muscle also increase significantly with activation^36–40^. In such circumstances, skeletal muscle thereby becomes a powerful competitor of the brain for glucose and oxygen^41^.

High intensity exercise increases the metabolic demand of skeletal muscles and the brain^39,40,42–45^, in proportion to degree of activation. At high levels of activation both are reliant upon glucose metabolism, and require a high rate of oxygen and glucose supply. Should both be challenged simultaneously, competition for these valuable yet limited resources may therefore develop, with one or both organs receiving an insufficient supply for optimal performance.

The concept of an antagonistic relationship between capacity to perform mental and physical work is not a new one^46^. As described by the idea of central fatigue, prior mental exertion may impair subsequent physical performance^47^.

Despite the intuitive appeal of a trade-off between two competing functions, negative covariance in such traits are not frequently observed when phenotypic comparisons are made between individuals within a population^48,49^. This study seeks to experimentally investigate the possibility of a trade-off involving the brain at the acute, rather than at the evolutionary or developmental, level. It is hypothesised that, when both systems are challenged simultaneously, performance will be inferior to performance when each are challenged in isolation. It is further hypothesised that the relative decrease in muscle power output will exceed the relative decrease in cognitive function.

## Results

A description of the samples is given in Table 1. Significant positive correlations were observed between rowing power output in both Protocols A and C (*r* = 0.484, *p* < 0.001), as well as recall performance in both Protocols B and C (*r* = 0.758, *p* < 0.001). This suggests that participants who row fast or recall many words do so irrespective of condition. Table 2 reports the correlation matrix (Pearsons product-moment correlation coefficients), with a significant negative correlation was observed between rowing power output in Protocol A and Δpower from Protocol A to C (*r* = −0.343, *p* = 0.006). No correlation was also observed between recall performance in Protocol B and Δrecall from Protocol B to C (*r* = −0.203, *p* = 0.113). This suggests that highly performing participants in Protocols A and B exhibit the greatest decline in performance when both tasks are performed together in Protocol C.

**Table 1:** Descriptive statistics for power output and free recall.

	Protocol A (n = 62)		Protocol B (n = 62)		Protocol C (n = 62)	
	M	SD	M	SD	M	SD
Power output (W)	389.93	34.819	—	—	340.20	43.321
Free recall (words)	—	—	29.11	3.339	26.27	3.738

Age M = 21.1 yrs, SD = 1.61; Weight M = 80.7 kg, SD = 4.46; Height M = 181.2cm, SD = 3.98.

**Table 2:** Correlation matrix.

	A: Power output	B: Recall	C: Power output	C: Recall	Δ Power	Δ Recall
A: Power output	—					
B: Recall	0.155	—				
C: Power output	0.484***	0.223	—			
C: Recall	0.102	0.758***	0.355**	—		
Δ Power output	−0.343**	0.105	0.656***	0.293*	—	
Δ Recall	−0.054	−0.203	0.234	0.485***	0.298*	—

Note: Statistical significance: *p < 0.05; **p < 0.01; ***p < 0.001.

**Figure 1:**
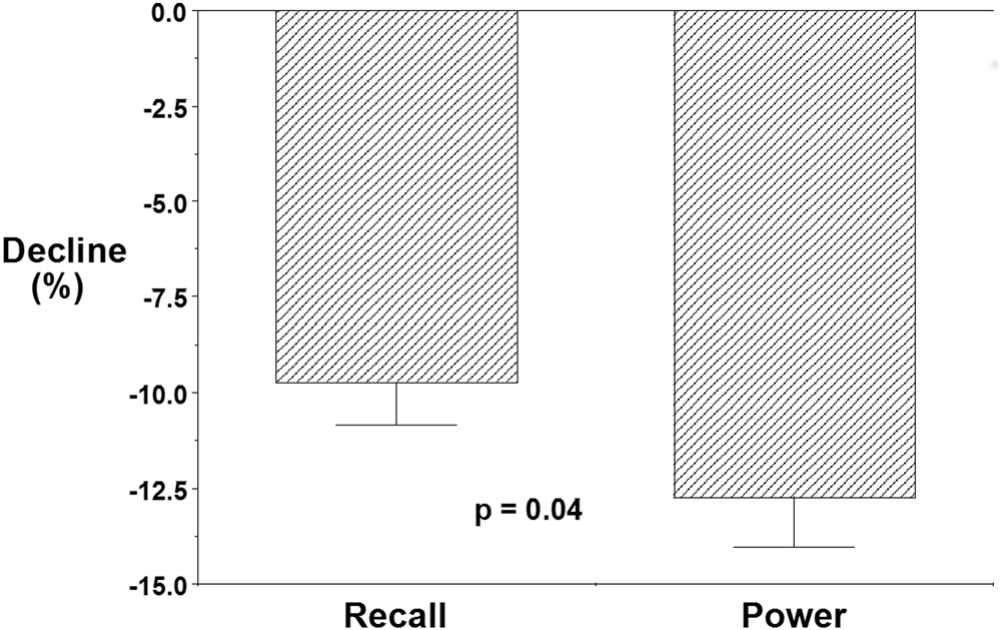
Bar graph showing the relative decrease in cognitive decrease and power output. Power output decreased significantly more than cognitive function.

Paired samples *t*-tests revealed that power output (W) was significantly lower in Protocol C than in Protocol A (Protocol A M = 389.93, SD = 34.819; Protocol C M = 340.20, SD = 43.321; *t*(61) = 9.699, *p* < 0.001). Similarly, recall (correct words) was significantly lower in Protocol C than in Protocol B (Protocol B M = 29.11, SD = 3.339; Protocol C M = 26.27, SD = 3.738; *t*(61) = 8.975, *p* < 0.001). The percentage change in recall between Protocols B and C was significantly less than the percentage change in power output between Protocol A and C (Δrecall M = −9.6740, SD = 8.62756; Δpower output M = −12.5535, SD = 9.81460, *t*(61) = −2.069, *p* = 0.043), see Fig. 1.

## Discussion

We proposed that the simultaneous challenge of both cognitive and physical faculties would result in impaired performance in each task, compared to performance achieved when in isolation. This hypothesis has been supported. The observation that both tasks cannot be performed optimally at the same time suggests that a trade-off between mental and physical function does indeed exist. The secondary hypothesis was supported as concurrent challenge differentially affected each task; the decrease in brain function was significantly less than that of power output.

Preferential allocation of glucose to the brain may be an evolved trait; a well-fuelled brain may offer better survival odds than well-fuelled muscles when facing an environmental challenge^31^. In such a situation, the body is able to buffer a muscle-fuel deficit by increasing supply of free fatty acids to fuel skeletal muscles^50,51^. High intensity physical activity considerably increases the metabolic needs of both the brain and skeletal muscle^28,38–40,42–44,52^. Competition for a limited supply of blood glucose and oxygen is a potential mechanism accounting for the fast-acting trade-off in brain and muscle function demonstrated here. The occurrence of glycogen supercompensation in the brain, as well as in skeletal muscles following exhaustive exercise, provides further support for this explanation^53^.

Although the brain is normally dependent upon glucose for energy, it may also utilise the lactate produced by skeletal muscles during exercise. The brain takes up lactate in proportion to its arterial concentration, which increases with exercise intensity. This increased lactate utilisation contributes to the meeting of high cerebral energy demands, which result from increased neuronal activity during high intensity exercise^54^. The preferential uptake of lactate as the predominant oxidative substrate of neurones^55^ has the effect of sparing glucose^56^. However, our results suggest that substrate competition between the brain and skeletal muscles is significant despite this.

Life history theory describes the competitive allocation of limited resources between physiological functions during development^57–60^. The optimal physiological distribution is determined by both the individual’s life stage^61^ and environment^62,63^, and is achieved through phenotypic plasticity^64^. Hales & Barker extended life history theory to consider trade-offs between organs and tissues, such as brain and muscle, by proposing that nutritional stress during early development leads to certain tissues being prioritised over others^65^. This tactic allows the organism to endure conditions of energy deficit, but with the cost of decreased adaptability to varying ecological conditions later in life due to decreased investment in the development in other organs.

During neonatal development, the brain is most vulnerable to irregularities in energy supply. The high adiposity of humans at birth in comparison to other mammalian species^66^ provides an energetic buffer^67^, preserving cerebral metabolism despite high early-life energetic requirements. Furthermore, preferential distribution of resources is evident in undernourished foetuses, in whom some organs grow normally while others are underdeveloped^68^. For humans, the brain’s development is spared^69^, perhaps at the expense of muscle^70^. Low birth weight, indicative of foetal undernourishment, is associated with a negative relationship between development of brain and muscles^71^. The selfish nature of the brain has also been observed in the unique preservation of brain mass in individuals suffering from long-term malnutrition or starvation^51^, in children born with intrauterine growth restriction^72,73^ and in glucose-challenging situations such as fasting or hypo/hyper-glycaemia^74–76^.

The evidence presented in this paper, which builds upon the existing body of research, indicates the possibility of an evolutionary trade-off between brain and muscle energetic demands.

## Conclusion

This study has demonstrated an acute level trade-off between cognitive function and physical power output during simultaneous challenge. This supports the selfish brain hypothesis due to the relative preservation of cognitive function over physical power output. The underlying mechanism is unclear, and requires further investigation.

## Methods

Sixty-two male rowers were recruited from the University of Cambridge, and testing was carried out in Cambridge, UK (mean age = 21.15 years, SD = 1.618 years). All participants were instructed in the risks and benefits of participating in the study and signed a written informed consent statement. The statement and the study was approved by the University of Cambridge Biology Ethics Committee (Application No: HBREC.2013.12), and the study was conducted in accordance with the approved methodology.

Participants completed three protocols (Table 3). Protocol A consisted of a maximum effort row for 3 minutes at free rate on a Concept 2 rowing ergometer (manufactured by Concept 2, Vermont, USA), and average power output (W) was recorded. Protocol B consisted of a mental free recall task, the number of words correctly recalled was recorded. Protocol C consisted of the same 3 minute row as A, but while simultaneously performing the mental task of Protocol B. Both average power output and number of words correctly recalled were recorded.

**Table 3:** Experimental protocols.

Experimental protocol summary	
Protocol	Description
A	Physical task
B	Mental task
C	Physical and mental task

The rowing ergometer was used because it is an energetically demanding activity, and has been used in previous studies investigating extreme physical stress^77,78^. The mental task involved free recall. A large printed screen showing 75 words was clearly displayed in front of the participants’ chair (Protocol B), or in front of the rowing ergometer (Protocol C), for a duration of 3 minutes. The participants were required to recall and write as many words as possible in any order from memory within 5 minutes (5 minutes immediately following the row in Protocol C)^79^. The words were selected from the Toronto Noun Pool^80^. Two 75-word lists were randomly created from the 150 words used by Kahana & Howard^81^ and were counterbalanced across participants. Half of the participants were given List 1 for Protocol B and List 2 for Protocol C, with the other half being given List 2 for Protocol B and List 1 for Protocol C. This method ensured that each word was seen an equal number of times across participants, and each participant saw each word only once. Such counterbalancing ensured that any artefacts^82^ were controlled for to reduce the likelihood of such artefacts^83^.

The Protocols were completed at 1 week intervals. All participants refrained from extra exercise the day before, and the day of, each Protocol. The same machine was used for Protocols A and B, with the drag factor being consistent. The order in which the participants completed the three protocols was also counterbalanced, in order to control for any effects such as the development of memorising strategies.
